# Peripheral cytokine and monocyte phenotype associations in drug-resistant epilepsy

**DOI:** 10.1038/s41598-025-14402-4

**Published:** 2025-08-13

**Authors:** Tracie Huey-Lin Tan, Richard P. Sequeira, Piero Perucca, Patrick Kwan, Terence J. O’Brien, Mastura Monif

**Affiliations:** 1 Department of Neuroscience, School of Translational Medicine, Faculty of Medicine, Nursing and Health Science, Monash University, Level 6 Alfred Centre, 99 Commercial Road, Melbourne, VIC 3004 Australia; 2 Department of Neurology, Alfred Hospital, Centre Block, Level 4, 55 Commercial Road, Melbourne, VIC 3004 Australia; 3 Department of Neurology, Royal Melbourne Hospital, 300 Grattan Street, Parkville, VIC 3052 Australia; 4 Bladin-Berkovic Comprehensive Epilepsy Program, Department of Neurology, Austin Health, 145 Studley Road, Heidelberg, VIC 3084 Australia; 5 Epilepsy Research Centre, Level 2, Melbourne Brain Centre, 245 Burgundy Street, Heidelberg, VIC 3084 Australia

**Keywords:** Drug resistant epilepsy, Psychogenic non-epileptic seizures, Cytokine, Chitinase 3-like 1, Monocyte, P2X7 receptor, Epilepsy, Neurology, Neuroscience, Diseases of the nervous system

## Abstract

Novel therapeutic targets are required to develop new treatments to lower the rates of drug-resistant epilepsy (DRE). This study assessed differences in plasma inflammatory biomarker concentrations and monocyte phenotype and function in patients with DRE versus psychogenic non-epileptic seizures (PNES). Luminex was used to analyse plasma samples from 21 DRE cases and 19 PNES controls for concentrations of selected cytokines and chitinase 3-like 1 (CHI3L1). Flow cytometry was used to quantify the percentage of monocytes expressing HLADR, CD14, CD16, CD11b, P2X7R and their median fluorescence intensity (MFI) ratio in 22 DRE and 11 PNES patients. Flow cytometry was used to assess P2X7 receptor (P2X7R) pore function via YO-PRO-1 uptake. No difference in cytokine and CHI3L1 concentrations was seen. Compared to PNES, DRE had a higher percentage of ‘classical’ monocytes (CD14++CD16−) and less ‘non-classical’ monocytes (CD14−CD16+). The percentage of ‘classical’ monocytes expressing P2X7R was increased in DRE compared to PNES. CD11b MFI ratio was increased in ‘classical’ and ‘intermediate’ (CD14+CD16+) monocytes. P2X7R pore function was similar between groups. Overall, while cytokine levels were similar between groups, the differences in monocyte phenotype indicates a more ‘proinflammatory’ circulating innate immune state in DRE. Thus, monocytes may be a novel therapeutic target for future research.

## Introduction

The rates of drug-resistant epilepsy (DRE) have not significantly altered over several decades despite the availability of numerous new antiseizure medications (ASMs)^1^. This highlights the need for novel treatment targets which in turn requires increased understanding of the pathogenesis of epilepsy. Neuroinflammation is one of the pathways found to be altered in both animal and human studies of epilepsy and seizures^2^. The role of immune mechanisms in human epilepsy differs depending on the underlying aetiology. It is a critical driver of acute symptomatic seizures secondary to autoimmune encephalitis^3^, but its role in more aetiologically diverse epilepsies is less clear. Numerous studies have shown alterations in protein and mRNA expression of various, mainly innate immunity related, cytokines and other inflammatory molecules (e.g. complement^4^ and inflammasome related proteins)^5^ as well as changes in microglial and monocyte phenotypes and function in non-autoimmune encephalitis associated epilepsies^6,7^. In particular, P2X7 receptor (P2X7R), a purinergic receptor activated by ATP and found on microglia as well as mononuclear cells, has been implicated in epileptogenesis via several mechanisms including activation of the NLRP3 inflammasome and production of interleukin 1β^8^.

This study examined for differences in peripheral soluble inflammatory biomarkers and monocyte phenotype and function in patients with DRE compared to control patients with psychogenic non-epileptic seizures (PNES; also known as “Functional Seizures”) without comorbid epilepsy. Plasma levels of various biomarkers involved in different ‘arms’ of the immune response, with a focus on innate immunity, was quantified and peripheral monocyte phenotype and activation (via P2X7R pore function) examined. PNES patients were selected as controls as they can mimic the physical phenotype of epileptic seizures but without abnormal electrical activity. While peripheral findings may not fully represent central nervous system pathology, studies utilising these samples add important complementary information to studies utilising brain tissue and cerebrospinal fluid^9^. Furthermore, any peripheral blood discoveries can be more readily translated for clinical use as diagnostic or prognostic biomarkers given samples are more easily accessible compared to cerebrospinal fluid or brain tissue.

We hypothesised that DRE would have a more ‘pro-inflammatory’ state than PNES with corresponding alterations in plasma cytokines and chitinase 3-like 1 (involved in injury response and mononuclear cell migration across the blood brain barrier (BBB)) and an increase in ‘pro-inflammatory’ or ‘classical’ monocyte phenotypes with greater P2X7R expression and pore function. Therefore, the study aims were to quantify plasma cytokine and chitinase 3-like 1 levels in DRE versus PNES patients and to assess monocyte phenotype and P2X7R pore function in these two groups.

## Materials and methods

### Study design and setting

This was a case–control study. The soluble biomarker arm utilised both prospectively and retrospectively collected data and the monocyte phenotype arm utilised prospectively collected data. Participants were recruited from two major tertiary hospitals in Melbourne, Australia (The Alfred Hospital and The Royal Melbourne Hospital). Data was stored in a password protected REDCap database. Blood samples were collected between March 2020 and July 2024 from patients admitted to the hospital as well as from patients attending clinical appointments. Luminex® testing was performed by TT and RS in a PC2 laboratory in Monash University, Australia. Flow cytometry was performed by TT utilising ARAFlowCore facilities, Monash University, Australia.

### Patient and clinical details

29 DRE and 22 PNES patients’ bloods were collected for monocyte flow cytometry analysis and/or plasma Luminex® multiplex assay. 14 DRE and eight PNES patients had both monocyte flow cytometry and plasma Luminex® multiplex assay performed. The remainder had either monocyte flow cytometry or plasma Luminex® multiplex assay.

All the DRE cohort met the ILAE diagnostic criteria for DRE^10^. Epilepsy was confirmed on video-EEG monitoring in all cases, following review by expert epileptologists. None had comorbid PNES. All PNES patients except for one had typical episodes confirmed on video-EEG monitoring and no PNES patients had evidence of comorbid epilepsy. Patient 20 had a normal EEG without a typical PNES event recorded, but a clinical presentation consistent with PNES. Cases and controls were excluded if they were taking immunomodulatory/immunosuppressive therapies, had a history of haematological malignancy or had an infection in the prior 2 weeks. All patients were inter-ictal at the time of blood collection, but time from last seizure was not recorded.

Demographic information and clinical details were obtained via review of medical records (retrospective data for PNES samples collected between March 2020 to Dec 2021) as well as interview of participants (all DRE samples and PNES samples collected between May 2021 to July 2024). The following information was obtained and stored in a redcap database: age at time of blood collection, sex, epileptic/non-epileptic seizure onset, seizure frequency score, current and past antiseizure medications (ASM), other medical diagnoses, the Neuropsychiatry Unit COGnitive Assessment Tool (NUCOG) score, radiological and nuclear medicine results.

### Definitions

Seizure frequency was converted to a seizure frequency score (SFS) ranging from 0 to 12 (Supplementary Table S1), as done in prior studies^11^. The score was used to quantify both epileptic seizure and PNES frequencies.

NUCOG is a cognitive screening tool developed by the Royal Melbourne Hospital, Australia. The total score has shown reliability and validity in epilepsy cohorts. It contains 25 questions testing: attention, visuoconstruction, memory, executive function and language with a total score out of 100^12^.

### Luminex® assay

Whole blood was centrifuged at 1500 g for 15 min and 600μL plasma aliquots stored at − 80 °C until use. Average times from blood draw to freezing was 95.7 min and 125.2 min in the DRE and PNES groups respectively. Samples were visually inspected for hemolysis and hemolysed samples were not used. Duration of sample storage prior to Luminex® multiplex assay were similar between the two groups. One plasma aliquot per patient was thawed and used for cytokine analysis (run in duplicate) (Supplementary Methods describes the materials and methods in more detail). A 14-PLEX custom designed Luminex® Discovery Assay for the detection of the following cytokines was utilized: tumour necrosis factor α (TNF-α), interleukin 6 (IL-6), interleukin 1β (IL-1β), interleukin 18 (IL-18), interleukin 10 (IL-10), interleukin 1 receptor antagonist (IL-1Ra), interleukin 8 (IL-8), C–C motif chemokine ligand 3 (CCL3), C–C motif chemokine ligand 2 (CCL2), C-X-C motif chemokine ligand 10 (CXCL10), C–C motif chemokine ligand 19 (CCL19), C-X-C motif chemokine ligand 9 (CXCL9) and chitinase 3-like 1 was used (Supplementary Table S2 shows plate design).

Concentrations of the assayed proteins was calculated by xPONENT software using a 5-parameter logistic regression curve. Percent coefficient of variation (%CV) was obtained for each duplicate and only those with a %CV < 20 were used for analysis. Any results below or above the lower limit (LLD) and upper limits of detection (ULD) were also removed from analysis. One DRE sample and one PNES sample were not included in IL-8 analysis due to being below the LLD; two DRE samples were not included in IL-1β analysis due to being below the LLD; one DRE and two PNES samples were not included in the IL-1Ra analysis as they were above the ULD; other excluded data points were due to unacceptable variability (i.e. %CV ≥ 20).

### Flow cytometry procedure

Monocytes were isolated from 30 to 40 mL of whole blood using an immunodensity negative selection antibody cocktail (Stemcell Technologies RosetteSep™ human monocyte enrichment cocktail) via density gradient centrifugation on Lymphoprep (Stemcell™ Technologies) (Supplementary Methods describes the materials and methods in greater detail).

For monocyte phenotyping, isolated monocytes were resuspended in 1 mL of phosphate buffered saline and 40μL aliquots of approximately 100,000 cells were stained with 1μL of fluorophore conjugated antibodies and diluted to 400 μL with eBioscience™ flow cytometry staining buffer, Invitrogen® (antibodies: anti-human HLADR, BD Biosciences®, cat no. 335796; anti-human CD16, Stemcell Technologies®, cat no. 60041PE; anti-human CD14, BD Biosciences®, cat no. 563079; anti-human CD11b, Stemcell Technologies, cat no. 60040; anti-human P2X7R, Sigma®, cat no. P8997). The cell suspension was then incubated for 20 min at 4 °C prior to centrifugation at 300 g for 5 min. The monocyte pellet was then resuspended in 500μL of flow cytometry staining buffer and flow cytometry performed.

For monocyte P2X7R functional testing, 40μL aliquots (as above) were made up to 540μL with 37 °C PBS (phosphate buffered saline, Gibco®, cat no. 10010023). AZ10606120 dihydrochloride (Sigma-Aldrich®, cat no. B6396) was added to make a final concentration of 4 μM in the appropriate tube. All samples were incubated for 15 min at 37 °C. Bz-ATP to a concentration of 200 μM (Tocris®, cat no. 3323-10 mg) and YO-PRO-1-iodide to a concentration of 5 μM (YO-PRO-1, Thermofisher®, cat no. Y3603) were added to the appropriate samples. Samples were again incubated at 37 °C for 30 min, then centrifuged at 300 g for 5 min. Monocyte pellets were resuspended in 500μL of flow cytometry staining buffer and flow cytometry performed.

**Fig. 1:**
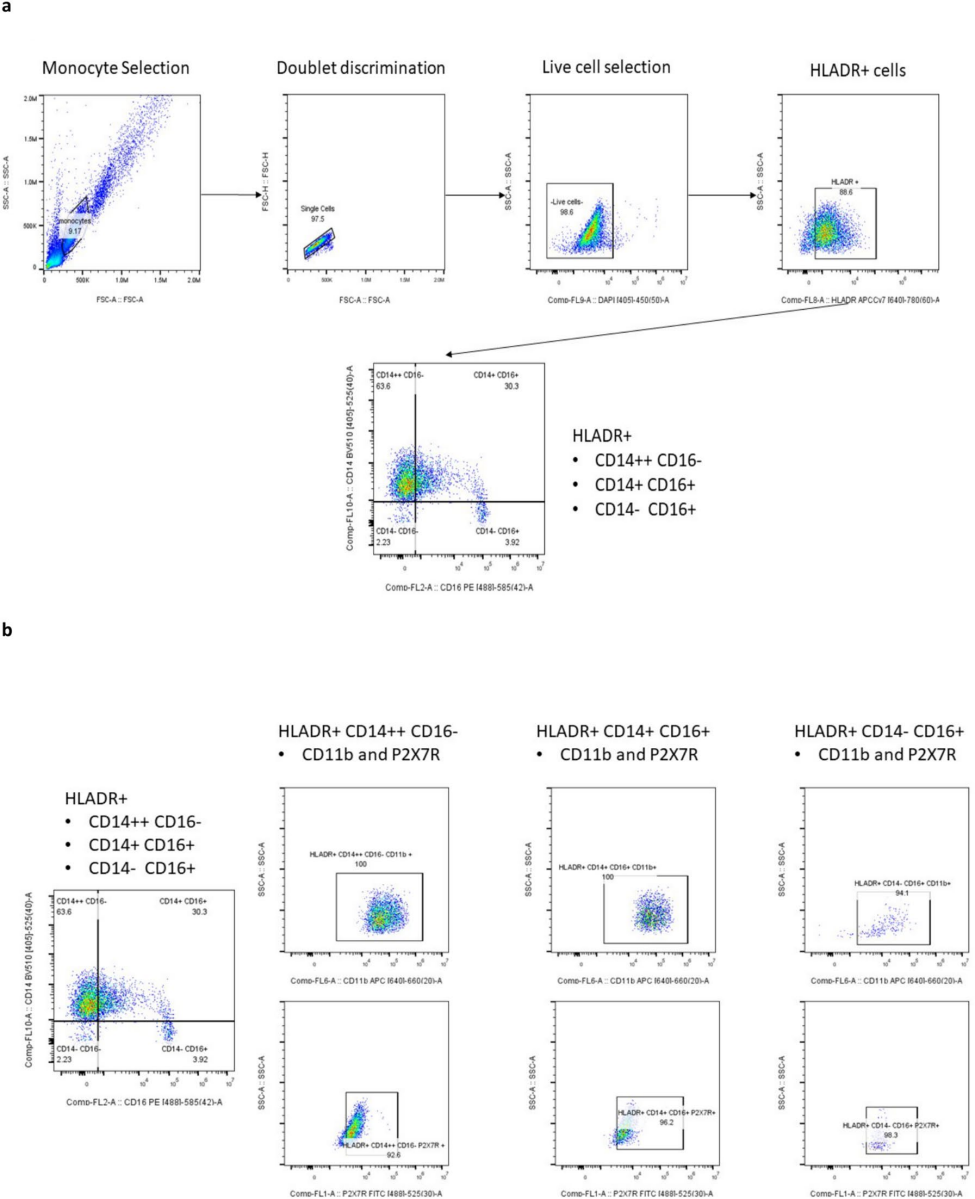
Monocyte gating strategy. (**a**) selection of live monocytes (DAPI negative) expressing HLADR and separation of those into classical (CD14++CD16−), intermediate (CD14+CD16+) and non-classical (CD14−CD16+) monocytes. (**b**) gating strategy for the selection of CD11b or P2X7R expression for each monocyte subset.

All live monocytes were assessed via flow cytometry recording 10,000 events per sample (Beckman Coulter CytoFLEX flow cytometer; CytExpert v2.4.0.28). Results were analysed using FlowJo version 10.8.1 using the gating strategies shown in Fig. 1. For the monocyte phenotype study, single stained live monocytes were used to adjust compensation using the ‘autospill’ option in FlowJo. The sample without antibodies added (‘unstained’) was used to define the minimum median fluorescence intensity (MFI) and the sample with all antibodies added (‘all stained’) was used to determine the maximum MFI for HLADR, CD11b, P2X7R, CD14 and CD16 signals. For the monocyte function study utilising YO-PRO-1 and P2X7R agonist (Bz-ATP) and antagonist (AZ10606120 dihydrochloride), a similar unstained sample was used to determine minimum MFI. MFI ratio was calculated from the following equation:

$${\text{MFI ratio}} = \left( {{\text{MFI}}_{{{\text{maximum}}}} {-}{\text{ MFI}}_{{{\text{minimum}}}} } \right)/{\text{MFI}}_{{{\text{minimum}}}}$$

Percentage of live monocytes expressing the various cell surface markers were also calculated from the ‘all stained’ sample. Cut offs for positive expression was gated using the single stained monocyte samples with unstained monocyte samples as a negative.

### Statistical analysis

Fisher’s exact test was used to assess categorical data. T-test or Mann–Whitney tests were used to assess continuous variables depending on distributional properties. The D’Agostino and Pearson test was used to test for normality. Multiple linear regression was used to assess the effect of potential confounders (age, sex) on the association between DRE or PNES status and monocyte phenotype. Spearman correlation was used to assess association between monocyte phenotype and number of ASMs used by DRE individuals. Simple linear regression was used to assess association between monocyte phenotype and sex. Statistical significance was set at *p* < 0.05. Analyses were performed with Graphpad Prism version 10.2.0.

### Ethics

This study was approved by the Human Research Ethics Committees of Alfred health (project no: 157/19 and 155/20) and Melbourne Health (project no: HREC/17/Alfred/168). All experiments were performed in accordance with the approved research protocol. All patients provided written informed consent with the approved patient information consent forms prior to participation in this study.

## Results

### Demographics and clinical information

Tables 1, 2 and 3 summarise the clinical and demographic information for the two groups.

**Table 1:** Drug resistant epilepsy cohort clinical and demographic details (part 1).

Case no	Age (yrs)	Sex	Epilepsy onset (age, yrs)	Diagnosis	SFS	No. of ASM	NUCOG	MRI	PET
1^pf^	36.8	F	31	Left temporal lobe epilepsy	8	3	92.5	No epileptogenic lesion	Normal
2^pf^	57.2	F	38	Bitemporal lobe epilepsy	9	1	82	No definite epileptogenic lesion	Normal
3^pf^	41.6	F	32	Multifocal epilepsy (past AIE)	7	2	70.5	Bilateral HS	–
4^pf^	26.2	F	25	Left frontal lobe epilepsy	7	3	–	No epileptogenic lesion	Normal
5^pf^	34	F	22	Left temporoinsular epilepsy	6	4	75	Focal volume loss left anterior insular cortex; left HS	Hypometabolism left insular cortex & mesial temp lobe
6^p^	36.3	M	20	Multifocal epilepsy	5	3	94	No epileptogenic lesion	–
7^pf^	33.5	F	26	Bitemporal lobe epilepsy (GAD Ab+)	7	3	87	Left HS	Hypometabolism left mesial temporal and temporal pole
8^pf^	21.5	F	20	Bitemporal lobe epilepsy (past NORSE)	7	2	–	No epileptogenic lesion	Bilateral temporal lobe hypometabolism
9^p^	36.5	F	35	Right temporal lobe epilepsy	10	1	–	Right amygdala enlargement with T2 hyperintensity	–
10^pf^	73.4	F	62	Bitemporal lobe epilepsy	9	2	75.5	No epileptogenic lesion	Hypometabolism bilateral mesial temporal lobes
11^pf^	38.5	F	35	Bitemporal lobe epilepsy (past AIE)	8	4	70.5	Bilateral HS	Normal
12^p^	44.2	M	24	Right hemispheric epilepsy	6	3	80.5	No epileptogenic lesion	Hypermetabolism right parietooccipital lobe
13^pf^	19.2	F	18	Multifocal epilepsy (FCD)	9	3	66.5	Left frontal atrophy, right prefrontal cortex T2 hyperintensity, right frontal T2 hyperintensity	Hypometabolism left frontal and right parietal metabolism, increased right frontal metabolism
14^pf^	78.6	F	73	Right temporal lobe epilepsy (post stroke)	7	2	68.5	Right corona radiata stroke	Normal
15^pf^	48.9	F	44	Left infraposterior frontal lobe epilepsy (post stroke)	7	2	94.5	Left posterior frontal encephalomalacia (stroke)	Hypometabolism left anterior mesial temporal lobe
16^pf^	24.4	M	19	Right temporal lobe epilepsy (encephalocele)	7	3	94	Right anterior temporal encephalocele	Hypometabolism right temporal pole

^p^Used for plasma Luminex® analysis; ^f^Used for flow cytometry analysis. Age refers to age at time of blood draw. Abbreviations: AIE, autoimmune encephalitis; ASM, antiseizure medication; F, female; FCD, focal cortical dysplasia; GAD Ab+, anti-glutamic acid decarboxylase antibody positive; HS, hippocampal sclerosis; M, male; MRI, magnetic resonance imaging; NORSE, new onset refractory status epilepticus; NUCOG, Neuropsychiatry Unit COGnitive screening tool score; PET, positron emission tomography; SFS, seizure frequency score.

**Table 2:** Drug resistant epilepsy cohort clinical and demographic details (part 2).

Case no	Age (yrs)	Sex	Epilepsy onset (age, yrs)	Diagnosis	SFS	No. of ASM	NUCOG	MRI	PET
17^pf^	64.1	M	48	Left temporal lobe epilepsy (encephalocele)	7	2	76.5	Multiple small bilateral temporal encephaloceles	Normal
18^p^	36.4	F	30	Bitemporal lobe epilepsy	7	4	86.5	No epileptogenic lesion	Hypometabolism left mesial temporal lobe
19^p^	27.1	M	23	Bitemporal lobe epilepsy	7	2	87	No epileptogenic lesion	Normal
20^p^	48	F	38	Bitemporal lobe epilepsy	6	4	80.5	Left greater than right HS	Hypometabolism left hippocampus
21^p^	25.4	F	18	Right insulo-opercular epilepsy (ganglioglioma)	8	5	98	Right posterior insular resection cavity residual ganglioglioma at the posterior aspect of resection cavity	Hypometabolism right anterior temporal lobe
22^f^	30.5	M	24	Right temporal lobe epilepsy (post-surgical gliosis)	7	4	99.5	Right HS; right inferior temporal pole encephalomalacia	Hypometabolism right temporal lobe
23^f^	41.1	M	4	Right temporal lobe epilepsy (LEAT)	8	2	–	Right temp fusiform gyrus LEAT	Hypometabolism right mesial temporal lobe
24^f^	65.2	M	2	Left frontal lobe epilepsy (FCD)	5	4	81	Left orbitofrontal region FCD	Hypometabolism left orbitofrontal region
25^f^	33.3	M	16	Left temporal lobe epilepsy (cavernoma)	9	5	94.5	Left anterior temporal lobe cavernoma; small right temporal lobe encephalocele	Hypometabolism anterior left temporal lobe
26^f^	28.9	M	13	Right temporal lobe epilepsy (likely amygdala MCD)	8	4	90	Right amygdala enlargement	Hypometabolism right mesial temporal lobe; subtle right orbitofrontal hypometabolism
27^f^	40.1	F	9	Right temporal lobe epilepsy (choroid plexus papilloma—WHO grade 1)	7	2	80.5	Right hippocampal lesion with calcification and focal enhancement	Hypometabolism right mesial temporal lobe
28^f^	23.1	F	14	Left temporal lobe epilepsy (DNET)	8	3	95	Mesial left temporal lobe DNET	Hypometabolism over left temporal lesion
29^f^	43.9	F	25	Right fronto-insular epilepsy (FCD)	9	3	99.5	No epileptogenic lesion	Equivocal hypometabolism right temporal pole and inferolateral temporal lobe

^p^Used for plasma Luminex® analysis; ^f^Used for flow cytometry analysis. Age refers to age at time of blood draw. Abbreviations: AIE, autoimmune encephalitis; ASM, antiseizure medication; DNET, dysembryoplastic neuroepithelial tumour; F, female; FCD, focal cortical dysplasia; HS, hippocampal sclerosis; LEAT, low grade epilepsy-associated neuroepithelial tumour; M, male; MCD, malformation of cortical development; MRI, magnetic resonance imaging; NUCOG, Neuropsychiatry Unit COGnitive screening tool score; PET, positron emission tomography; SFS, seizure frequency score.

**Table 3:** Psychogenic non-epileptic seizure cohort clinical and demographic details.

Control no	Age (years)	Sex	Seizure onset (age, years)	SFS	no. of ASM#	NUCOG	MRI
1^pf^	23.6	F	23	8	0	92	No epileptogenic lesions
2^pf^	66.2	F	65	9	1	–	No epileptogenic lesions
3^pf^	42.5	F	41	8	1	73.5	No epileptogenic lesions
4^pf^	27.5	F	19	8	0	94.5	No MRI
5^pf^	27.7	F	22	10	0	90	No epileptogenic lesions
6^pf^	25.1	F	14	8	0	94.5	No epileptogenic lesions
7^pf^	32.9	F	24	8	1	86.5	No epileptogenic lesions
8^pf^	22.1	M	18	6	1	57	No epileptogenic lesions
9^p^*	37.1	M	33	8	3	97.5	No epileptogenic lesions
10^p^*	20	F	16	8	0	84	No epileptogenic lesions
11^p^*	51.9	F	45	7	0	98.5	No MRI
12^p^*	62.4	F	58	8	1	97	Encephalomalacia in bilateral inferior frontal lobes and left temporal lobe. Small area of gliosis left insular cortex (infarct)
13^p^*	45	F	44	9	0	81	No epileptogenic lesions
14^p^*	44	F	43	9	2	90	No epileptogenic lesions
15^p^*	33.9	F	18	5	2	100	No epileptogenic lesions
16^p^*	37	F	29	7	3	87.5	No epileptogenic lesions
17^p^*	32.5	F	31	8	1	92.5	No epileptogenic lesions
18^p^*	29.2	F	29	9	0	91	No epileptogenic lesions
19^p^*	28.7	F	24	7	1	79	No MRI
20^f^	32.3	F	13	9	0	–	No epileptogenic lesions
21^f^	51.2	F	26	9	1	–	No epileptogenic lesions
22^f^	40.7	F	37	3	0	–	No epileptogenic lesions

^f^Used for flow cytometry analyses; ^p^Used for plasma Luminex® analysis;*clinical data collected retrospectively (others prospectively). Age refers to age at time of blood draw. Abbreviations: ASM, anti-seizure medication; F, female; M, male; MRI magnetic resonance imaging; NUCOG, Neuropsychiatry Unit COGnitive screening tool score; SFS, seizure frequency score.

### Plasma Luminex® multiplex assay experiment—demographic and clinical information

Plasma samples from 21 DRE cases and 19 PNES controls were included for Luminex® analysis. Mean age of DRE cases at blood draw was similar to PNES controls (40.6 ± 16.4 versus 36.3 ± 12.99 years, *p* = 0.37). 76.2% of DRE patients and 89.5% of PNES controls were female (*p* = 0.41). Of note, two PNES controls were female-to-male transgender and were classified by sex (female).

Both groups had high seizure (epileptic or non-epileptic) frequencies with mean(± SD) SFS of 7.3 (± 1.2) in the DRE group and 7.9 (± 1.2) in the PNES group. The number of ASM prescribed per patient was higher in the epilepsy group with a mean(± SD) of 2.8 (± 1.0) medications compared to 0.9 (± 1.0) in the PNES group (*p* < 0.0001). PNES controls were on ASM for reasons including prior misdiagnosis of epilepsy, migraines, mood disorders and neuropathic pain. Cognitive performance appeared slightly lower based on NUCOG testing in the epilepsy group with a median score of 81.3 (IQR 73.9–92.9) versus 90.5 (IQR 83.3–95.1) in the PNES group, although this did not reach statistical significance (*p* = 0.06).

### Flow cytometry—demographic and clinical information

Monocytes were isolated from 22 DRE patients and 11 PNES controls for flow cytometry. Mean age of DRE patients at blood draw was 41.1 years (95%CI 33.6–48.6 years) which was similar to PNES controls (35.6 years, 95%CI 26.5–44.7 years, *p* = 0.36). 68.2% of DRE patients were female compared to 90.9% of PNES controls, *p* = 0.21. Of these, 20 DRE cases and 10 PNES controls also had P2X7R pore function tested.

The flow cytometry groups also had high seizure burden with a mean SFS of 7.0 (IQR 7.0–8.3) in the DRE group and 8.0 (IQR 8.0–9.0) in the PNES group, *p* = 0.19. Again, the number of ASM being taken by the DRE group was higher than that of the PNES controls (median: 3, IQR 2–4 versus 0.0, IQR 0–1 respectively, *p* < 0.0001). Cognitive performance, where measured, was similar between the two groups with median NUCOG scores of 82 (IQR 75.0–94.5) in the DRE group versus 90 (IQR 73.5–94.5) in the PNES group (*p* = 0.97).

### Plasma Luminex® multiplex assay results

**Fig. 2:**
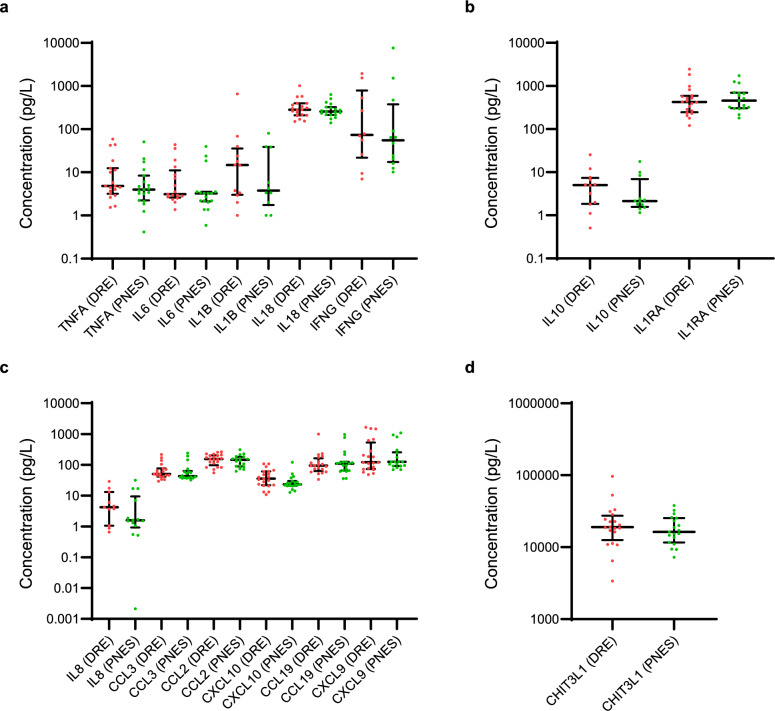
Concentrations of various analytes in picograms/litre. Results obtained using Luminex to test plasma from drug resistant epilepsy patients and psychogenic non-epileptic controls—there were no differences found between the two cohorts. (**a**) ‘proinflammatory’ cytokines; (**b**) ‘immunomodulatory’ cytokines; (**c**), chemokines; (**d**), Chitinase-3 Like 1 protein. Abbreviations: DRE, drug resistant epilepsy; PNES, psychogenic non-epileptic seizures.

Fourteen proteins were assayed and there was no difference in any of the analyte levels between DRE patients and PNES controls. ‘Pro-inflammatory’ cytokines (TNF-α, IL-6, IL-1β and IL-18), ‘immunomodulatory’ cytokines (IL-10 and IL-1Ra), chemokines (IL-8, CCL3, CCL2, CXCL10, CCL19 and CXCL9) and chitinase-3 like-1 protein levels are demonstrated in Fig. 2 and Supplementary Table S3.

### Monocyte phenotypes in DRE versus PNES

**Fig. 3:**
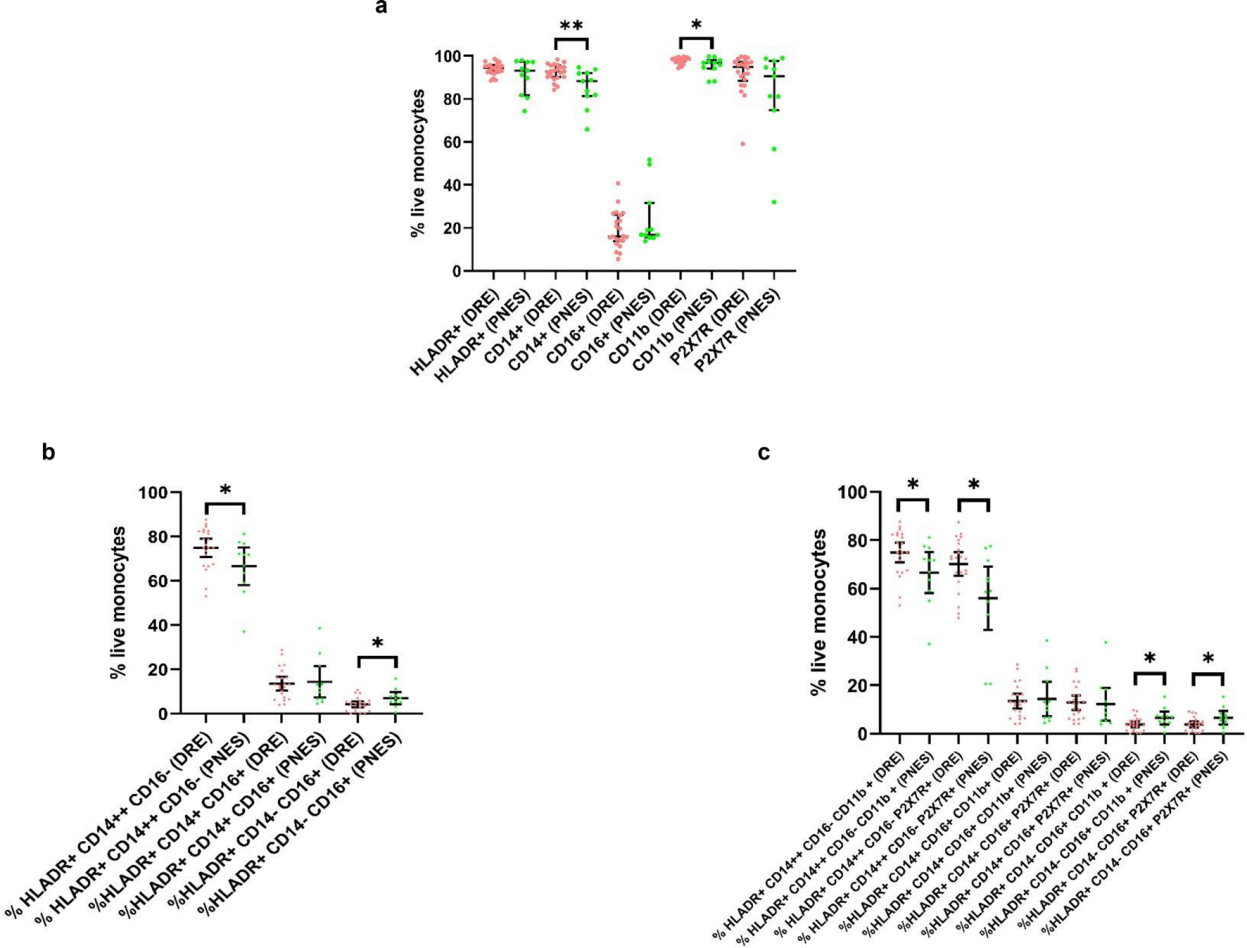
Percentage of live monocytes expressing various cell surface markers. Median and interquartile ranges depicted. *p > 0.01 to 0.05, **p 0.001 to ≤ 0.01. (**a**) percentage of live monocytes expressing single cell surface markers; (**b**) percentage of HLADR positive live monocytes with classical (CD14++CD16−), intermediate (CD14+CD16+) and non-classical (CD14− CD16+) phenotypes; (**c**) percentage of subsets in b that express CD11b or P2X7R. Abbreviations: DRE, drug resistant epilepsy; PNES, psychogenic non-epileptic seizures.

There were more monocytes expressing CD14, in line with a higher percentage of classical monocytes and a lower number of ‘non-classical’ monocytes, in the DRE group versus the PNES group (Supplementary Table S4, Fig. 3, panel A and B). CD11b, a marker of monocyte activation, was also expressed by more monocytes in the DRE compared to PNES group (Supplementary Table S4, Fig. 3, panel A). Differences in the percentage of ‘classical’ and ‘non-classical’ monocytes expressing CD11b reflected the overall prevalence of each subtype (Supplementary Table S4, Fig. 3, panel C). P2X7R expression was disproportionately higher in the ‘classical’ monocytes from the DRE compared to the PNES group (Supplementary Table S4, Fig. 3, panel C).

**Fig. 4:**
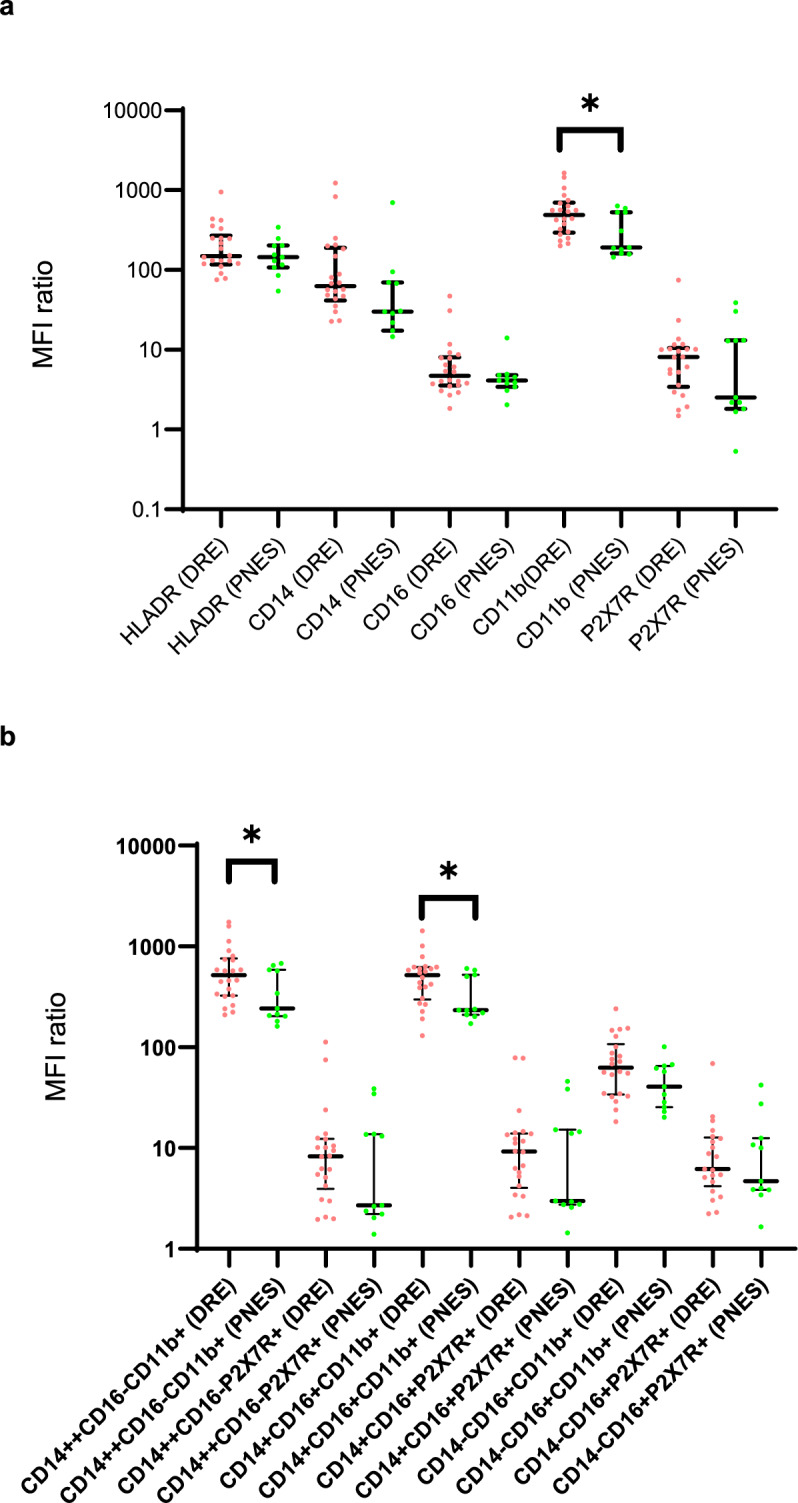
Median fluorescence intensity (MFI) ratios for various cell surface markers. MFI ratio = (MFI_all stained sample_ − MFI_unstained sample_)/MFI_unstained sample_. Results are displayed on a log10 scale. Median and interquartile range are shown. *p > 0.01 to 0.05. (**a**) MFI ratio of live monocytes expressing single cell surface markers; (**b**) MFI ratio of HLADR positive live monocytes with classical (CD14++CD16−), intermediate (CD14+CD16+) and non-classical (CD14− CD16+) phenotypes. Abbreviations: DRE, drug resistant epilepsy; PNES, psychogenic non-epileptic seizures.

The median fluorescence intensity (MFI) ratio of CD11b was greater in the monocytes of the DRE compared to the PNES group which may reflect the higher percentage of CD11b positive cells in the DRE group or increased expression of CD11b (Supplementary Table S5, Fig. 4, panel A). Furthermore, there was a higher CD11b MFI ratio in ‘classical’ and ‘intermediate’ monocyte subtypes of DRE patients suggesting an increased expression of this marker by these monocytes compared to PNES patient ‘classical’ and ‘intermediate’ monocytes (Supplementary Table S5, Fig. 4, panel B). P2X7R MFI ratios were similar between the two groups.

### P2X7R pore function in DRE patient derived monocytes versus PNES

**Fig. 5:**
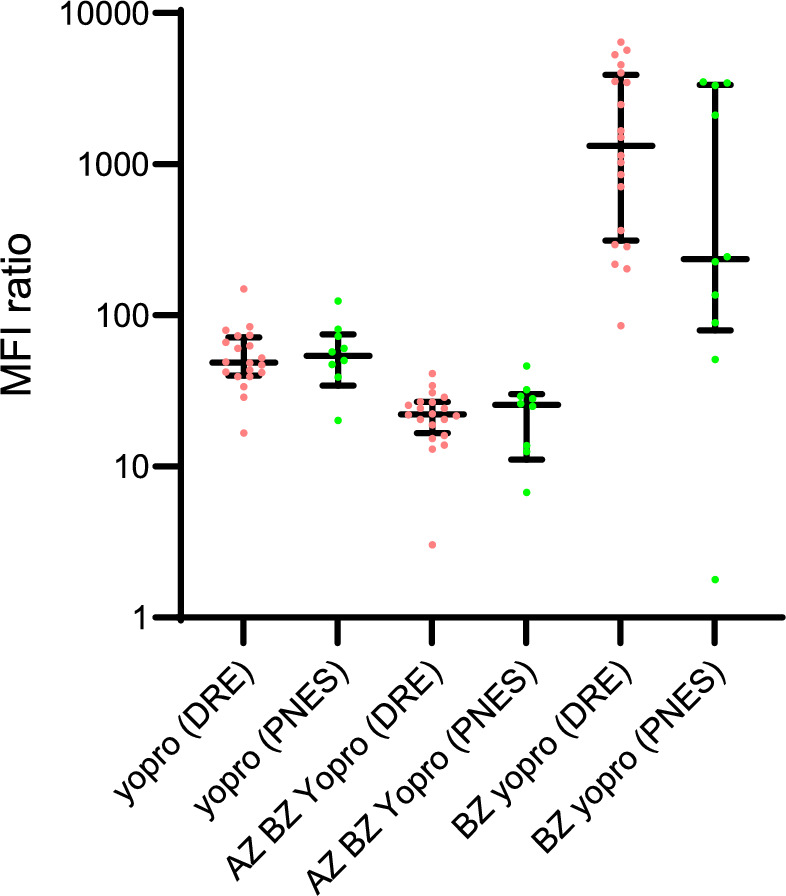
Monocyte P2X7R functional pore assay comparison between drug resistant epilepsy and psychogenic non-epileptic seizure cohorts. Median fluorescence intensity ratio of monocytes treated with YO-PRO-1 Iodide (5 µM) with or without concomitant exposure to Bz-ATP (P2X7R agonist; 200 µM) and/or Az (P2X7R antagonist, 4 µM). Abbreviations: AZ, AZ 10606120 dihydrochloride; BZ, 2’(3’)-O-(4-Benzoylbenzoyl)adenosine 5’-triphosphate triethylammonium salt; DRE, drug resistant epilepsy; PNES, psychogenic non-epileptic seizure; YO-PRO, YO-PRO-1 Iodide.

There was no difference in P2X7R pore function between the two groups. Baseline uptake of YO-PRO-1 was similar in untreated live monocytes between the two groups. Treatment of cells with Bz-ATP, a P2X7R agonist, caused an increase in YO-PRO-1 uptake in monocytes in both groups and its action was no different between groups (Supplementary Table S6, Fig. 5). This effect was reversed by pre-treatment with AZ10606120 (P2X7R antagonist; 4 µM) prior to treatment with Bz-ATP (200 µM) supporting a P2X7R mediated mechanism of YO-PRO-1 transport into monocytes. Furthermore, the uptake of YO-PRO-1 was reduced with AZ10606120 pre-treatment compared to baseline YO-PRO-1 to a similar extent in both DRE and PNES groups.

### Confounders in flow cytometry results

When accounting for age and sex, percentage of ‘classical’ monocytes and non-‘classical’ monocytes remained significantly positively (β 9.4, 95%CI 1.6–17.2, *p* = 0.02) and negatively (β − 3.2, 95%CI − 5.8 to − 0.6, *p* = 0.02) correlated with DRE respectively. There was no correlation between number of ASM or sex and percentage of ‘classical’ or ‘non-classical’ monocytes in the DRE group.

## Discussion

The first part of this study utilised Luminex® multiplex assay to quantify 14 inflammation related proteins (cytokines, chemokines and chitinase-3 like 1) in the plasma of DRE patients versus PNES controls. Several peripheral biomarkers of inflammation have been shown to be altered in patients with aetiologically diverse DRE supporting their use in research as a surrogate measure of neuroinflammation. For instance, IL-18^13^, interferon gamma, IL-1β^14–16^ and IL-6^15^ show increase in plasma or serum over healthy controls reflecting mRNA findings in epileptic brain tissue^16^. However, studies are variable with some showing no change or even a decrease in peripheral levels of IL-1β or IL-6 compared healthy controls^17,18^. Furthermore, the correlation between central (brain; cerebrospinal fluid, CSF) and peripheral (blood) inflammatory markers is imperfect. A study looking at serum and CSF cytokines in children with prolonged febrile seizures found discordant cytokine levels between the two sampling methods with IL-6 levels higher in CSF than serum and IL-10 and soluble TNF receptor 1 (sTNFR1) higher in serum than CSF^19^. Therefore, easily obtainable, peripheral samples are not perfect surrogates for determining central pathology. In line with the variability in findings between studies, there was no difference in the ‘pro-inflammatory’ cytokines TNFα, IL-6, IFN gamma, nor was there any difference in the inflammasome related cytokines IL-1β, IL-18 between our two groups. IL-10 and IL-1Ra, which are more ‘immunomodulatory’ cytokines previously found to be increased^20^ or decreased^21^ in plasma from children with febrile seizures compared to those with fevers and no seizures, was also no different between DRE and PNES patients.

Various chemokines have also been studied in relation to epilepsy and seizures. CCL2, a key monocyte/macrophage chemoattractant^22^, and CCL3, a macrophage, polymorphonuclear cell, B and CD8 T lymphocyte chemoattractant^23^, have been most studied and serum and plasma levels of these chemokines are increased in patients with epilepsy compared to healthy or non-epileptic controls^24,25^. Peripheral blood mRNA transcripts of IL-8, a neutrophil chemoattractant and activator^26^, was upregulated in DRE^27^ and serum levels were increased^25^ compared to healthy controls, but unchanged in plasma compared to non-epileptic controls^24^. CXCL10 and CXCL9 are T cell and monocyte/macrophage chemoattractants and are involved in promoting Th1 polarisation^28,29^. They have been less studied in epilepsy, but were increased in the CSF of febrile infection-related epilepsy syndrome (FIRES) patients presenting with status epilepticus compared to comparators with epilepsy^30,31^ with no difference between those with epilepsy and non-inflammatory neurological controls^31^. Similarly, CCL19, a chemokine involved in adaptive immunity and T cell migration^32^, was elevated in the CSF of FIRES compared with epilepsy patients with minimal elevation in epilepsy patients compared to non-inflammatory neurological controls^31^. In this study, no difference between chemokine plasma levels in DRE and PNES patients were identified.

Levels of chitinase 3-like 1, a protein involved in injury response and mononuclear cell transmigration across the BBB^33,34^, was also analysed. It has not been studied in epilepsy, but is elevated in the CSF of multiple sclerosis, an inflammatory CNS disease, and correlates with disease activity^35,36^. However, it has also been found to be less sensitive when measured in serum with no difference in levels between multiple sclerosis groups and healthy controls^34^. Similarly, the levels of chitinase 3-like 1 protein in plasma was similar between our two groups.

Overall, given the variability of past studies in relation to blood cytokine and chemokine alterations and the potentially lower sensitivity in general when measuring biomarker levels in peripheral samples in the setting of CNS disease, the lack of differential levels of cytokines between groups in this study was unsurprising. This suggests that these are insensitive biomarkers of epilepsy and that there is, at most, low levels of peripheral inflammation occurring in DRE, although this likely varies based on aetiology. The dynamic alterations of the above markers, their responsiveness to various therapies, and their temporal relationship to seizure events, all need further assessment.

The second part of this study involved analysing peripheral monocyte phenotype and function in DRE compared to PNES. Seizures can induce BBB disruption^37,38^ and, in concert with increased chemokine production centrally (e.g. CCL2), attract monocytes into the CNS^39^. In animal studies, these infiltrating monocytes, along with their microglial counterparts, contribute to microgliosis and neuronal cell death following kainic acid induced seizures^40,41^. Furthermore, blocking CCL2 action (either with CCL2 neutralising antibody or by using CCR2 knock out mice) resulted in reduced monocyte/macrophage infiltration^41,42^. This in turn resulted in lower kainic acid induced neuronal cell death and a lower seizure severity on kainic acid re-exposure^41^. In human epilepsy, infiltrating peripherally derived antigen presenting cells (including monocytes) were increased in brain tissue of patients with epilepsy and correlated with seizure severity and frequency^43^. Therefore, CNS infiltrating monocytes may play a role in epileptogenesis and/or ictogenesis.

There was a greater proportion of ‘classical’ monocytes (CD14++CD16−) and lower levels of ‘non-classical’ monocyte (CD14− CD16+) subsets in the DRE group compared to the PNES group. ‘Classical’ monocytes display phagocytic properties, produce reactive oxygen species and pro-inflammatory molecules (e.g. IL-6 and IL-8) and express higher levels of chemokine receptors allowing them to migrate efficiently towards injury and participate in acute inflammatory responses^44,45^. ‘Non-classical’ monocytes do not produce pro-inflammatory cytokines to the same extent as ‘classical’ monocytes and are involved in antigen presentation^46^. There was no difference in ‘intermediate’ monocyte (CD14+CD16+) subsets between groups. ‘Intermediate’ monocytes have a function in between ‘classical’ and ‘non-classical’ monocytes and are involved in antigen presentation and can produce pro-inflammatory and anti-inflammatory cytokines^44,47,48^.

Compared to PNES, DRE patients had a higher percentage of monocytes expressing activation marker CD11b and this increase was attributable to an increase in percent of ‘classical’ monocytes (CD14++CD16−) expressing this marker. In line with an increased number of monocytes expressing CD11b, MFI ratio was also higher in DRE compared to the PNES. Furthermore, the CD11b MFI ratio was higher in ‘classical’ (CD14++CD16−) and intermediate (CD14+CD16+) monocytes of DRE suggesting that expression was increased in these monocyte subsets compared to those from PNES. CD11b is involved in cellular adhesion and transmigration across endothelial cells into target tissues^49^. Increased CD11b monocyte expression levels has been reported in REM sleep behavioural disturbance and Parkinson’s disease with higher expression correlating with cognitive impairment in the latter disease^50,51^.

Percentage of monocytes expressing the P2X7R, another marker of activation, was similar between groups (95% DRE versus 90.5% PNES), but more ‘classical’ monocytes expressed P2X7R in the DRE compared to the PNES group making up 70.2% versus 56% of monocytes respectively. However, the P2X7R MFI ratio of the ‘classical’ monocytes was similar between groups. This suggests that while more DRE monocytes express P2X7R, the level of expression at the individual cell level is similar between groups. Of note, P2X7R is expressed on activated monocytes in response to ATP binding and leads to a pro-inflammatory response that includes the release of cytokines such as IL-1β and IL-18^52^. Past studies have found upregulation in P2X7R protein levels in human temporal lobe epilepsy brain samples compared to postmortem non-neurological controls and elevation in the plasma levels of patients with temporal lobe epilepsy compared to healthy controls and PNES patients^53–55^. Overall, the prior literature suggests that activated peripheral monocytes may play a role in the pathophysiology of epilepsy and drug resistance and the results of this study are consistent with that.

Finally, P2X7R pore activity was investigated. YO-PRO-1 baseline uptake into monocytes through the P2X7R was similar between the DRE and PNES monocytes. This baseline uptake was reduced upon exposure to a P2X7R antagonist (AZ10606120) even with subsequent agonist (Bz-ATP) exposure. This reduction in baseline uptake was similar between the two groups. Exposure to agonist alone (Bz-ATP), resulted in a similar increase in YO-PRO-1 uptake in both groups. This suggests that the pore activity of the P2X7R between groups is similar at the individual cell level.

This study utilised human samples which allows for generation of clinically relevant results and is a major strength of this study. Furthermore, the analysis of peripheral monocyte phenotypes has not been extensively investigated in the epilepsy population, and in particular P2X7R function has not been studied. Thus, this study provides novel insights into the peripheral innate immune function in DRE patients. Our study utilised PNES patients as a control group, a disease considered to have a psychiatric basis. The strength of this method was that PNES patients more closely mimic the physical stressors that are experienced by epilepsy patients during seizures than healthy controls. Furthermore, they are often misdiagnosed as epilepsy patients and therefore any differences in findings between groups could also be used as a diagnostic biomarker if validated in the future. However, given that they are not completely ‘healthy’, it is difficult to know whether the similarities between groups is due to shared inflammatory changes or truly ‘normal’ results. For instance, PNES patients often have comorbid psychiatric conditions such as depression, post traumatic stress disorder and anxiety^56,57^, diseases that have been reported to be associated with alterations in circulating cytokine levels^58,59^. This may explain some of the negative cytokine findings. Furthermore, a reasonable number of data points were excluded due to high variability between duplicates during analysis of cytokines. This limited the power of the study and may have contributed to negative findings. There was also high intragroup variability and therefore a larger number of participants in both the groups is likely required to detect changes. The time from last epileptic seizure was not recorded and this may have contributed to the high intragroup variability. However, the variability in the PNES groups appeared generally similar apart from IL-6 (and to a lesser extent, IFN gamma, CXCL10 and CXCL9) which have less variability in the PNES group. IL-6 is increased in the post-ictal period with only variable changes in the interictal period in past studies suggesting it is a biomarker of recent seizure activity rather than epilepsy^17,60,61^. Nevertheless, despite the inclusion of patients whom may have had analyte levels artificially raised from baseline by being taken within 24 h of last seizure (but still in the interictal period), there were no significant differences in analyte levels between groups.

Future studies utilising a healthy control group, drug responsive epilepsy patients, larger numbers and taking into account time from last seizure will be required to confirm and potentially expand the findings of this study. Furthermore, given the changes in cytokine levels in the periphery and CNS are not always consistent due to BBB impermeability, additional studies investigating the cytokine profiles of DRE patients in cerebrospinal fluid would be useful to better delineate the role of neuroinflammation in DRE. Given that P2X7R expression was increased on peripheral ‘classical’ monocytes, it would also be useful to further quantify soluble P2X7R levels (as conducted previously^62^) in the two groups. Also, given the similarities in P2X7R pore function as measured by YO-PRO-1 uptake, further alternative studies into P2X7R functioning (e.g. calcium conductance properties; P2X7R channel responses) would be useful to better elucidate functional change. Proteomic studies and RNA sequencing, along with intracellular cytokine staining of monocytes would also be useful to further define functional alterations occurring in DRE.

Overall, this study showed no significant differences in plasma cytokines and chemokines between DRE and PNES patient groups. Given that peripheral inflammation may not accurately reflect the processes occurring centrally, this does not negate the possibility of central neuroinflammation playing a role in DRE pathophysiology. On the other hand, peripheral monocyte phenotypes did differ between the two groups with a greater percentage of ‘classical’ monocytes, traditionally thought of as the ‘pro-inflammatory’ monocyte, and less ‘non-classical’ monocytes in the DRE group. The DRE classical monocytes were also more likely to express CD11b and P2X7R indicating a more activated state. Overall, this study supports the idea that pro-inflammatory peripheral immune alterations occur in DRE patients when compared to PNES controls. Whether this contributes to central neuroinflammation or epilepsy pathophysiology cannot be determined from this study and future studies are required to build on these findings. Nevertheless, this study provides a first stepping stone towards the understanding of the role of the peripheral innate immune system, in particular classical monocytes, in DRE. Of note, there exists several investigational and clinical therapies that are already in production that target neuroinflammation. Future studies providing a more granular understanding of central nervous system cytokine alterations and P2X7R function will be required prior to repurposing of such treatments for DRE.

## Supplementary Information

Supplementary Information 1.

Supplementary Information 2.

Supplementary Information 3.

Supplementary Information 4.

Supplementary Information 5.

Supplementary Information 6.

Supplementary Information 7.

## Data Availability

The datasets generated during and/or analysed during the current study are not publicly available due to the inclusion of confidential patient information. However, unpublished de-identified data will be made available from the corresponding author on reasonable request.
