# Supplementary material for: Peripheral cytokine and monocyte phenotype associations in drug-resistant epilepsy
Source: Sci Rep. 2025 Aug 13;15:29654. doi: 10.1038/s41598-025-14402-4 (PMC12350764; doi:10.1038/s41598-025-14402-4)
Supplement: Supplementary file 1 — Supplementary Information 1. [file 41598_2025_14402_MOESM1_ESM.docx]

**SUPPLEMENTARY METHODS**

**ELISA procedure**

ELISA Kit:

| Kit Name | Analyte | Company | Catalogue Number | Lot Number |
| --- | --- | --- | --- | --- |
| Luminex Discovery Assay | TNF-α  IL-6  IL-1β  IL-18  IL-10  IL-1ra  IL-8  CCL3  CCL2  CXCL10  CCL19  CXCL9  Chitinase 3 like 1 | R&D systems | LXSAHM-14 | L153781 |

Reagents:

| **Product Name** | **Company** | **Catalogue number** |
| --- | --- | --- |
| Hydrochloric acid, 36% | Univar | AJA1367-2.5LGL |
| Sodium hydroxide pellets | Sigma-Aldrich | S5881-500G |
| HEPES (1M) | Gibco | 15630-080 |
| Deionised water | - | - |
| Ethanol 100% undenatured | Chem-supply | EL043-10L-P |
| Bleach | - | - |
| Luminex® 100/200 Claibration Kit | Luminex | LX2R-CAL-K25 |
| Luminex® 100/200 Performance Verification Kit | Luminex | LX2R-PVER-K25 |

Equipment:

| **Product Name** | **Company** |
| --- | --- |
| Multipette® E3 | Eppendorf |
| Combitips advanced®, 10ml | Eppendorf |
| Combitips advanced®, 2.5ml | Eppendorf |
| Stripette® Serological pipette, 25mL | Corning® Costar® |
| Stripette® Serological pipette, 50mL | Corning® Costar® |
| S1 Pipet Filler | Thermo Scientific^TM^ |
| Acura 825 Pipette, 20-200uL | Socorex |
| Eppendorf® Research® Pipette, 100-1000uL | Eppendorf |
| Filtered pipette tips, 1mL | Abdos |
| Filtered pipette tips, 200uL | Abdos |
| 500mL bottle | - |
| 15ml Centrifuge Tube | Corning |
| 50mL Centrifuge Tubes | Corning |
| PCR® tubes, 500uL | Axygen |
| MTS 2/4 digital microtitre shaker | IKA |
| Personal vortex mixer | Ratek |
| Luminex^TM^ 200^TM^ Instrument System | Luminex |
| 50TS microplate washer | Biotek |
| 96-well Ring Magnet | Biotek |
| Microfuge® 20 centrifuge | Beckman Coulter |

ELISA Procedure:

Samples (plasma/supernatant) were thawed from the -80^o^C freezer in which they were stored and processed in a PC2 (Physical Containment Level 2) certified molecular laboratory as noted above. Luminex or Procartaplex ELISA kits as outlined in table 3-4 were used and manufacturer’s protocol followed apart from the following deviations which were performed to increase the ELISA signal:

1. Samples were used neat (undiluted)
2. Microparticle cocktail (Luminex kits) / magnetic beads (Procartaplex kits) were added to each sample as per protocol but incubated overnight on a plate shaker at 4^o^C (8-12 hours) instead of 2 hours at room temperature.
3. Microplate was allowed to sit on the magnet prior to washing for 2 minutes instead of 30 seconds to 1 minute.
4. Washing was performed twice instead of three time.

Samples were then read on a Luminex^TM^ 200.

**Monocyte isolation procedure**

Reagents:

| **Product Name** | **Company** | **Catalogue Number** |
| --- | --- | --- |
| RosetteSep™ Human Monocyte Enrichment Cocktail | StemCell Technologies | 15068 |
| UltraPure^TM^ 0.5M ethylenediaminetetraacetic acid (EDTA), pH8.0 | Invitrogen | 15575-038 |
| Lymphoprep™ density gradient medium | StemCell Technologies | 07851 |
| Sterile 1x phosphate-buffered saline, pH 7.4 | Gibco | 10010023 |

Equipment:

| Eppendorf® Research® Pipette, 100-1000uL | Eppendorf |
| --- | --- |
| Acura 825 Pipette, 20-200uL | Socorex |
| Filtered pipette tips, 1mL | Abdos |
| Filtered pipette tips, 200uL | Abdos |
| Stripette® Serological pipette, 25mL | Corning® Costar® |
| LabServ®Transfer pipette, 1mL | Thermofisher |
| S1 Pipet Filler | Thermo Scientific^TM^ |
| Microtubes, 1.7mL | Axygen |
| 5 Roller Tube Roller Mixer (BTR5-12V) | Ratek |
| Avanti J-15R Centrifuge | Beckman Coulter |
| JS-4.750 Swinging-bucket Rotor and Grey Buckets | Beckman Coulter |
| SafeMate ECO plus 1.5 Class 2 Biological Safety Cabinet | Laftech |
| Microfuge® 20 centrifuge | Beckman Coulter |

Monocyte isolation procedure

Monocyte isolation was conducted in a PC2 certified tissue culture laboratory in a HEPA laminar flow tissue culture cabinet (SafeMate ECO plus 1.5 Class 2 Biological Safety Cabinet). Appropriate personal protective equipment (mask, gown, gloves, eye wear, fully enclosed foot wear) was used. Sterile technique was observed in the tissue culture hood to prevent contamination of samples. This required the use of: 80% ethanol spray for decontamination of materials and gloves, sterile materials and filtered pipette tips were used in the biosafety cabinet.

Blood a biohazardous waste generated during sample processing was treated with 10% sodium hypochlorite or alternative suitable chemical disinfectant prior to disposal. Solid waste products were disposed of in the appropriate yellow biohazard bags/sharps bins and the hood cleaned with 80% ethanol prior to UV decontamination following use.

Blood for monocyte isolation was collected via venepuncture into EDTA tubes (aiming a total of around 30-40mL). The procedure is outlined as follows:

1. Whole blood was transferred from EDTA tubes into 2 x 50mL centrifuge tubes (approx. 15-20mL per tube)
2. 100μL of 0.5M EDTA and 600μL RosetteSep^TM^ human monocyte cocktail was added to each 50mL centrifuge tube
3. 50mL centrifuge tubes containing blood, EDTA and RosetteSep^TM^ human monocyte cocktail were then placed on a 5 roller tube roller mixer for 30 minutes at room temperature (speed set to ‘3’)
4. After 30 minutes, each 50mL centrifuge tube was topped up to 25mL with sterile room temperature PBS
5. Two new 50mL centrifuge tubes were prepared with 15mL of lymphoprep^TM^ density gradient medium added to each tube using a 25mL serological pipette and S1 Pipet filler. Care was taken to avoid getting any onto the sides of the centrifuge tube.
6. Using another 25mL serological pipette and S1 Pipet Filler, the blood from step 4 was gently layered on top of the Lymphoprep^TM^ density gradient medium making sure that the blood remained separate from the medium.
7. The lids of each tube was closed securely and the tubes transferred to the Avanti J-15R centrifuge without disturbing the two discrete layers
8. The samples were centrifuged for 25 minutes at 2000rpm (rotations per minute) with break off (i.e. acceleration ramp 10, deceleration ramp 1) at room temperature.
9. After centrifugation, a transfer pipette was used to transfer the layer between the plasma and Lymphoprep^TM^ that contained the isolated monocytes into a new 50mL centrifuge tube.
10. The centrifuge tube was then topped up with sterile PBS (to 50mL)
11. The centrifuge tube containing the monocyte suspension was transferred to the Avanti J-15R centrifuge and spun at 1600rpm for 10 minutes at room temperature (acceleration ramp 9, deceleration ramp 3).
12. After centrifugation, the supernatant (lymphoprep^TM^/plasma) covering the monocyte pellet was aspirated and discarded, ensuring the pellet remained undisturbed
13. The monocyte pellet was resuspended with 1mL of sterile PBS

**Flow cytometry procedure**

Reagents:

Antibody-fluorophore conjugates for labelling of monocytes prior to flow cytometry:

| **Antibody** | **Clone** | **Conjugate** | **Isotype** | **Manufacturer** | **Catalogue Number** |
| --- | --- | --- | --- | --- | --- |
| Anti human HLADR | L243 | APC-Cy7 | Mouse IgG2a, κ | BD Biosciences | 335796 |
| Anti human CD16 | 3G8 | PE | Mouse monoclonal IgG1, κ | Stemcell Technologies | 60041PE |
| Anti human CD14 | MφP9 | BV510 | Mouse BALB/c IgG2b, κ | BD Biosciences | 563079 |
| Anti human CD11b | ICRF44 | APC | Mouse monoclonal IgG1, κ | Stemcell Technologies | 60040 |
| Anti human P2X7R | polyclonal | FITC | Rabbit | Sigma | P8997 |
| Rabbit IgG-FITC isotype | Polyclonal | FITC | IgG | abcam | ab37406 |

YO-PRO-1 experimental reagents for the treatment of isolated monocytes prior to flow cytometry:

| **Product Name** | **Company** | **Catalogue number** |
| --- | --- | --- |
| YO-PRO-1 Iodide (1mM) | ThermoFisher | Y3603 |
| AZ 10606120 dihydrochloride  (P2X7R antagonist) | Tocris | 3323-10mg |
| 2’(3’)-O-(4-Benzoylbenzoyl)adenosine 5’-triphosphate triethylammonium salt (Bz-ATP)  (P2X7R agonist) | Sigma-Aldrich | B6396 |

General reagents:

| **Product Name** | **Company** | **Catalogue Number** |
| --- | --- | --- |
| eBioscience^TM^ Flow Cytometry Staining Buffer | Invitrogen | 00-4222-26 |
| DAPI ready-made solution, 1mg/mL  (for assessment of cell viability via flow cytometry) | Sigma | MBD0015-10mL |

Equipment:

| **Product Name** | **Company** |
| --- | --- |
| Microtubes, 1.7mL | Axygen |
| Eppendorf® Research® Pipette, 100-1000uL | Eppendorf |
| Acura 825 Pipette, 20-200uL | Socorex |
| Acura 825 Pipette 0.5-10uL | Socorex |
| Filtered pipette tips, 1mL | Abdos |
| Filtered pipette tips, 200uL | Abdos |
| Filtered pipette tips, 10uL | Abdos |
| Microfuge® 20 centrifuge | Beckman Coulter |
| Euroclone® Safegrow Pro, 5%CO2, 95%O2 | Laftech |
| Precision GP 20 water bath | Thermoscientific |
| Accublock^TM^ mini digital dry bath | Labnet |
| Cytoflex Flow Cytometer | Beckman Coulter |

Procedure for monocyte flow cytometry:

Labelling of monocytes with fluorophore conjugated antibodies:

Isolated monocytes were labelled with fluorescent antibodies and examined via flow cytometry as follows:

1. 1.7mL microtubes were labelled with a prespecified tube number according to antibody to be added
2. 40μL aliquots of monocyte suspension were pipetted into each labelled 1.7mL microtube
3. For tubes containing monocyte suspension:
   1. 1μL of the corresponding antibody (apart from DAPI) was added to the 40μL monocyte suspension. 0.5μL of DAPI was added just prior to running samples on the flow cytometer to the appropriate cells.
   2. Monocyte containing tubes were topped up with 360μL of flow buffer and mixed with pipette or vortex.
   3. Tubes were incubated at 4^o^C for 20 minutes (protected from light).
   4. Samples were then centrifuged in the microfuge® centrifuge at 300g for 5 minutes
   5. The supernatant was removed and monocyte pellet resuspended with 500μL of flow buffer
   6. Samples were then taken for flow cytometry. DAPI was added just prior to performing flow on appropriate DAPI requiring samples.

For monocyte P2X7 receptor function testing:

1. PBS was placed into water bath and warmed to 37^o^C
2. 500μL of 37^o^C PBS was added to each of the 4 microtubes containing 40μL monocyte suspension
3. AZ10606120 (final concentration 4µM) was added into the appropriate tube
4. All microtubes were incubated at 37^o^C for 15 minutes
5. BzATP (final concentration of 200μM) and YO-PRO-1 (5μM final concentration) was added into the corresponding tubes
6. Microtubes were incubated for 30 minutes at 37^o^C
7. Microtubes were centrifuged following incubation at 300g for 5 minutes
8. Supernatant was removed and cell pellet resuspended in 500μL of flow buffer
9. Flow cytometry was then performed

Procedure used for making Bz-ATP and AZ 10606120 dihydrochloride

Bz-ATP, 5mg vial (molecular weight: 715.39g/mol):

1. 500μL PBS was added to 5mg Bz-ATP and mixed to yield a concentration of 0.0139M
2. 15μL was aliquoted into separate microtubes and placed on dry ice immediately
3. All tubes were stored in a zip lock bag and labelled with name, concentration, date and store in a -20^o^C freezer until use
4. Individual aliquots were defrosted prior to use
   1. 7.5μL Bz-ATP into 500μL of solution yielded a concentration of 200μM (used in this research)

AZ10606120 dihydrochloride (molecular weight: 540.52g/mol)

1. 2mL of DMSO was added to 2g of AZ10606120 to give a concentration of 0.00185M
2. 16.5uL was aliquoted into separate microtubes and placed on dry ice immediately
3. All tubes were stored in a zip lock bag and labelled with name, concentration, date and stored in a -20^o^C freezer until use
4. Individual aliquots were defrosted prior to use
   1. 1μL of AZ10606120 into 500μL of solution will yield a concentration of around 4μM (used in this research)
