# Supplementary material for: Peripheral cytokine and monocyte phenotype associations in drug-resistant epilepsy
Source: Sci Rep. 2025 Aug 13;15:29654. doi: 10.1038/s41598-025-14402-4 (PMC12350764; doi:10.1038/s41598-025-14402-4)
Supplement: Supplementary file 2 — Supplementary Information 2. [file 41598_2025_14402_MOESM2_ESM.docx]

**Supplementary Table S1**. Seizure frequency score

| Seizure frequency | Score |
| --- | --- |
| Seizure-free, no ASMs  Seizure-free, need for ASMs unknown  Seizure-free, needs ASMs  Non-disabling simple partial seizures  Non-disabling nocturnal seizures only  Number of disabling seizures  1–3 per year  4–11 per year  1–3 per month  1–6 per week  1–3 per day  4–10 per day  >10 per day  Status epilepticus | 0  1  2  3  4  5  6  7  8  9  10  11  12 |
