# Supplementary material for: Peripheral cytokine and monocyte phenotype associations in drug-resistant epilepsy
Source: Sci Rep. 2025 Aug 13;15:29654. doi: 10.1038/s41598-025-14402-4 (PMC12350764; doi:10.1038/s41598-025-14402-4)
Supplement: Supplementary file 3 — Supplementary Information 3. [file 41598_2025_14402_MOESM3_ESM.docx]

**Supplementary Table S2.** Luminex® plate layout

|  | 1 | 2 | 3 | 4 | 5 | 6 | 7 | 8 | 9 | 10 | 11 | 12 |
| --- | --- | --- | --- | --- | --- | --- | --- | --- | --- | --- | --- | --- |
| **A** | standard 1 | standard 1 | Case 1 | Case 1 | Control 5 | Control 5 | Case 10 | Case 10 | Control 13 | Control 13 | Case 18 | Case 18 |
| **B** | standard 2 | standard 2 | Control 1 | Control 1 | Case 5 | Case 5 | Control 9 | Control 9 | Case 14 | Case 14 | Control 17 | Control 17 |
| **C** | standard 3 | standard 3 | Case 2 | Case 2 | Control 6 | Control 6 | Case 11 | Case 11 | Control 14 | Control 14 | Case 9 | Case 9 |
| **D** | standard 4 | standard 4 | Control 2 | Control 2 | Case 8 | Case 8 | Control 10 | Control 10 | Case 15 | Case 15 | Control 18 | Control 18 |
| **E** | standard 5 | standard 5 | Case 3 | Case 3 | Control 7 | Control 7 | Case 12 | Case 12 | Control 15 | Control 15 | Case 19 | Case 19 |
| **F** | standard 6 | standard 6 | Control 3 | Control 3 | Case 6 | Case 6 | Control 11 | Control 11 | Case 16 | Case 16 | Control 19 | Control 19 |
| **G** | standard 7 | standard 7 | Case 4 | Case 4 | Control 8 | Control 8 | Case 13 | Case 13 | Control 16 | Control 16 | Case 20 | Case 20 |
| **H** | universal buffer | universal buffer | Control 4 | Control 4 | Case 7 | Case 7 | Control 12 | Control 12 | Case 17 | Case 17 | Case 21 | Case 21 |
