# Supplementary material for: Peripheral cytokine and monocyte phenotype associations in drug-resistant epilepsy
Source: Sci Rep. 2025 Aug 13;15:29654. doi: 10.1038/s41598-025-14402-4 (PMC12350764; doi:10.1038/s41598-025-14402-4)
Supplement: Supplementary file 4 — Supplementary Information 4. [file 41598_2025_14402_MOESM4_ESM.docx]

**Supplementary Table S3.** Plasma concentrations (picograms/litre) of various analytes as measured by Luminex®

| Analyte (N, number used in analysis) | Median concentration (pg/mL) | IQR (pg/mL) | P Value |
| --- | --- | --- | --- |
| ‘Proinflammatory’ Cytokines | | | |
| TNF-α (DRE, N= 19) | 4.83 | 3.18 – 12.44 | 0.34 |
| TNF-α (PNES, N=19) | 3.98 | 2.24 – 8.40 |  |
| IL-6 (DRE, N=17) | 3.11 | 2.59 – 11.05 | 0.29 |
| IL-6 (PNES, N=17) | 3.26 | 2.12 – 3.55 |  |
| IL-1β (DRE, N=12) | 14.82 | 3.03 – 35.70 | 0.47 |
| IL-1β (PNES, N=10) | 3.78 | 1.75 – 38.84 |  |
| IL-18 (DRE, N=19) | 281.50 | 209.40 – 396.90 | 0.60 |
| IL-18 (PNES, N=19) | 256.2 | 215.10 – 326.00 |  |
| IFN gamma (DRE, N=10) | 73.96 | 21.79 – 782.80 | 0.63 |
| IFN gamma (PNES, N=12) | 55.55 | 17.25 – 377.00 |  |
| ‘Immunomodulatory’ Cytokines | | | |
| IL-10 (DRE, N=12) | 5.02 | 1.86 – 7.38 | 0.41 |
| IL-10 (PNES, N=12) | 2.13 | 1.58 – 6.92 |  |
| IL-1ra (DRE, N=20) | 425.60 | 246.10 – 589.90 | 0.54 |
| IL-1ra (PNES, N=17) | 456.90 | 303.40 – 698.90 |  |
| Chemokines | | | |
| IL-8 (DRE, N=11) | 4.21 | 1.05 – 13.18 | 0.30 |
| IL-8 (PNES, N=14) | 1.58 | 0.92 – 9.45 |  |
| CCL3 (DRE, N=19) | 50.38 | 41.61 – 76.85 | 0.39 |
| CCL3 (PNES, N=18) | 43.01 | 37.71 – 62.88 |  |
| CCL2 (DRE, N=21) | 153.40 | 97.79 – 205.30 | 0.59 |
| CCL2 (PNES, N=18) | 144.40 | 89.14 – 183.10 |  |
| CXCL10 (DRE, N=21) | 35.68 | 21.80 – 60.86 | 0.08 |
| CXCL10 (PNES, N=19) | 23.11 | 21.02 – 29.70 |  |
| CCL19 (DRE, N=20) | 94.67 | 62.58 – 162.70 | 0.65 |
| CCL19 (PNES, N=19) | 109.50 | 65.36 – 125.00 |  |
| CXCL9 (DRE, N=20) | 121.00 | 73.24 – 535.2 | 0.94 |
| CXCL9 (PNES, N=18) | 125.40 | 91.58 – 258.1 |  |
| Chitinase 3-like 1 | | | |
| Chitinase 3-like 1 (DRE, N=20) | 18997 | 12510 – 27467 | 0.43 |
| Chitinase 3-like 1 (PNES, N=19) | 16269 | 11635 – 25294 |  |

Abbreviations: DRE, drug resistant epilepsy; IQR, interquartile range; PNES, psychogenic non-epileptic seizures
