# Supplementary material for: Peripheral cytokine and monocyte phenotype associations in drug-resistant epilepsy
Source: Sci Rep. 2025 Aug 13;15:29654. doi: 10.1038/s41598-025-14402-4 (PMC12350764; doi:10.1038/s41598-025-14402-4)
Supplement: Supplementary file 5 — Supplementary Information 5. [file 41598_2025_14402_MOESM5_ESM.docx]

**Supplementary Table S4.** Percentage of the live monocyte subset expressing the various cell surface markers.

| **Cell surface marker**  **(DRE, N= 22; PNES, N=11)** | **Median or (mean) % live monocytes** | **IQR or (95%CI)** | **P value** |
| --- | --- | --- | --- |
| **Single cell surface markers** | | | |
| HLADR+ (DRE) | (94.0) | (92.8 – 95.3) | 0.07 |
| HLADR+ (PNES) | (90.4) | (85.0 – 95.9) |  |
| CD14+ (DRE) | (92.5) | (90.8 – 94.1) | **0.002** |
| CD14+ (PNES) | (85.2) | (79.3 – 91.2) |  |
| CD16+ (DRE) | (19.1) | (15.3-22.9) | 0.20 |
| CD16+ (PNES) | (24.2) | (14.9-33.6) |  |
| CD11b+ (DRE) | (98.0) | (97.3 – 98.6) | **0.01** |
| CD11b+ (PNES) | (95.4) | (92.7 – 98.1) |  |
| P2X7R+ (DRE) | 95.0 | 88.4 – 97.2 | 0.19 |
| P2X7R+ (PNES) | 90.5 | 74.8 – 97.7 |  |
| **HLADR+ cell subsets: classical, intermediate, non-classical monocytes** | | | |
| CD14++ CD16- (DRE) | 75.7 | 67.4 – 82.4 | **0.04** |
| CD14++ CD16- (PNES) | 71.8 | 59.1 – 76.9 |  |
| CD14+ CD16+ (DRE) | (13.5) | (10.5 – 16.6) | 0.77 |
| CD14+ CD16+ (PNES) | (14.4) | (7.3 – 21.6) |  |
| CD14- CD16+ (DRE) | (4.2) | (2.9 – 5.6) | **0.04** |
| CD14- CD16+ (PNES) | (7.0) | (4.3 – 9.7) |  |
| **HLADR+ CD14++ CD16- (classical monocytes): CD11b or P2X7R expression** | | | |
| CD11b+ (DRE) | 75.7 | 67.4 – 82,4 | **0.04** |
| CD11b+ (PNES) | 71.8 | 59.1 – 76.9 |  |
| P2X7R+ (DRE) | (70.2) | (65.4 – 75.0) | **0.01** |
| P2X7R+ (PNES) | (56.0) | (42.9 – 69.2) |  |
| **HLADR+ CD14+ CD16+ (intermediate monocytes): CD11b or P2X7R expression** | | | |
| CD11b+ (DRE) | (13.5) | (10.4 – 16.6) | 0.77 |
| CD11b+ (PNES) | (14.4) | (7.2 – 21.6) |  |
| P2X7R+ (DRE) | (12.9) | (9.8 – 15.9) | 0.82 |
| P2X7R+ (PNES) | (12.2) | (5.4 – 18.9) |  |
| **HLADR+ CD14- CD16+ (non-classical monocytes): CD11b and P2X7R expression** | | | |
| CD11b+ (DRE) | (3.9) | (2.7 – 5.1) | **0.03** |
| CD11b+ (PNES) | (6.6) | (3.9 – 9.2) |  |
| P2X7R+ (DRE) | (3.9) | (2.7 – 5.1) | **0.03** |
| P2X7R+ (PNES) | (6.6) | (3.8 – 9.4) |  |

Median and interquartile range is given for results analysed using Mann Whitney test and mean and standard deviation given for results analysed using a t-test. Abbreviations: 95%CI, 95% confidence interval; DRE, drug resistant epilepsy; IQR, interquartile range; PNES, psychogenic non-epileptic seizures
