# Supplementary material for: Peripheral cytokine and monocyte phenotype associations in drug-resistant epilepsy
Source: Sci Rep. 2025 Aug 13;15:29654. doi: 10.1038/s41598-025-14402-4 (PMC12350764; doi:10.1038/s41598-025-14402-4)
Supplement: Supplementary file 6 — Supplementary Information 6. [file 41598_2025_14402_MOESM6_ESM.docx]

**Supplementary Table S5.** Median fluorescence intensity (MFI) ratios for various cell surface markers.

| **Cell surface marker**  **(DRE, N=22^*^; PNES, N=11)** | **Median MFI ratio** | **IQR** | **P value** |
| --- | --- | --- | --- |
| **Single cell surface marker MFI ratio on live monocytes** | | | |
| HLADR+ (DRE) | 148.2 | 117.5 – 270.9 | 0.46 |
| HLADR+ (PNES) | 144.6 | 107.3 – 202.1 |  |
| CD14+ (DRE) | 62.6 | 41.6 – 188.4 | 0.32 |
| CD14+ (PNES) | 29.9 | 18.5 – 88.3 |  |
| CD16+ (DRE) | 4.7 | 3.6 – 8.1 | 0.40 |
| CD16+ (PNES) | 4.1 | 3.4 – 4.8 |  |
| CD11b+ (DRE) | 490.1 | 294.6 – 698.1 | **0.02** |
| CD11b+ (PNES) | 190.8 | 160.5 – 530.6 |  |
| P2X7R+ (DRE) | 8.1 | 3.4 – 10.6 | 0.72 |
| P2X7R+ (PNES) | 2.5 | 1.8 – 13.1 |  |
| **HLADR+ CD14++ CD16- (classical monocytes): CD11b or P2X7R MFI ratio** | | | |
| CD11b+ (DRE) | 517.4 | 324.3 – 760.1 | **0.048** |
| CD11b (PNES) | 243.0 | 204.1 – 587.2 |  |
| P2X7R+ (DRE) | 8.3 | 3.9 – 12.3 | 0.64 |
| P2X7R+ (PNES) | 2.7 | 2.2 – 13.7 |  |
| **HLADR+ CD14+ CD16+ (intermediate monocytes): CD11b or P2X7R MFI ratio** | | | |
| CD11b+ (DRE) | 520.3 | 298.0 – 623.1 | **0.04** |
| CD11b (PNES) | 233.3 | 210.5 – 521.8 |  |
| P2X7R+ (DRE) | 9.2 | 4.0 – 14.0 | 0.67 |
| P2X7R+ (PNES) | 3.0 | 2.7 – 15.1 |  |
| **HLADR+ CD14- CD16+ (non-classical monocytes): CD11b and P2X7R MFI ratio** | | | |
| CD11b+ (DRE) | 63.0 | 34.0 – 107.6 | 0.11 |
| CD11b (PNES) | 40.7 | 25.5 – 65.1 |  |
| P2X7R+ (DRE)^*^ | 6.2 | 4.2 – 12.7 | 0.78 |
| P2X7R+ (PNES) | 4.7 | 3.9 – 12.6 |  |

MFI ratio = (MFI_stained sample_ – MFI_unstained_ _live monocytes_)/ MFI_unstained_ _live monocytes_. ^*^N = 21 due to no cells of this subset in one sample to ascertain an MFI. Abbreviations: DRE, drug resistant epilepsy; IQR, interquartile range; PNES, psychogenic non-epileptic seizures
