# Supplementary material for: Peripheral cytokine and monocyte phenotype associations in drug-resistant epilepsy
Source: Sci Rep. 2025 Aug 13;15:29654. doi: 10.1038/s41598-025-14402-4 (PMC12350764; doi:10.1038/s41598-025-14402-4)
Supplement: Supplementary file 7 — Supplementary Information 7. [file 41598_2025_14402_MOESM7_ESM.docx]

**Supplementary Table S6.** Monocyte P2X7R functional pore assay.

| **YO-PRO-1 ± Az/Bz**  **(DRE, N=20;**  **PNES, N=10)** | **Median or (mean) MFI ratio** | **IQR or (95%CI)** | **P value** |
| --- | --- | --- | --- |
| **DRE versus PNES** | | | |
| YO-PRO (DRE) | 48.7 | 40.1 – 71.6 | >0.99 |
| YO-PRO (PNES) | 53.8 | 34.2 – 75.0 |  |
| YO-PRO + Az + Bz (DRE) | (22.5) | (18.6 – 26.3) | 0.89 |
| YO-PRO + Az + Bz (PNES) | (21.9) | (12.0 – 31.8) |  |
| YO-PRO + Bz (DRE) | (2196) | (1220 – 3172) | 0.25 |
| YO-PRO + Bz (PNES) | (1316) | (181.6 – 2450) |  |
| **DRE cohort** | | | |
| YO-PRO (DRE) | 48.7 | 40.1 – 71.6 | **<0.0001** |
| YO-PRO + Bz (DRE) | 1324 | 312.6 – 3915 |  |
| YO-PRO (DRE) | 48.7 | 40.1 – 71.6 | **<0.0001** |
| YO-PRO + Az + Bz (DRE) | 22.2 | 16.7 – 26.8 |  |
| YO-PRO + Bz (DRE) | (2196) | (1220 - 3172) | **<0.0001** |
| YO-PRO + Az + Bz (DRE) | (22.5) | (18.6 – 26.3) |  |
| **PNES cohort** | | | |
| YO-PRO (PNES) | (55.3) | (30.9 – 79.6) | **0.02** |
| YO-PRO + Bz (PNES) | (1316) | (181.6 – 2450) |  |
| YO-PRO (PNES) | (55.3) | (30.9 – 79.6) | **0.01** |
| YO-PRO + Az + Bz (PNES) | (21.9) | (12.0 – 31.8) |  |
| YO-PRO + Bz (PNES) | (1316) | (181.6 – 2450) | **0.02** |
| YO-PRO + Az + Bz (PNES) | (21.9) | (12.0 – 31.8) |  |

Median fluorescence intensity ratio of monocytes treated with YO-PRO-1 Iodide (5µM) with or without concomitant exposure to Bz-ATP (P2X7R agonist; 200µM) and/or Az (P2X7R antagonist, 4µM). Abbreviations: AZ, AZ 10606120 dihydrochloride; BZ, 2’(3’)-O-(4-Benzoylbenzoyl)adenosine 5’-triphosphate triethylammonium salt; DRE, drug resistant epilepsy; IQR, interquartile range; PNES, psychogenic non-epileptic seizure; yopro, YO-PRO-1 Iodide
